# Acute myocardial infarction and pulmonary embolism in a young man with pernicious anemia-induced severe hyperhomocysteinemia

**DOI:** 10.1186/1477-9560-7-5

**Published:** 2009-05-13

**Authors:** Ayyash Melhem, Ankit Desai, Marion A Hofmann

**Affiliations:** 1 Department of Medicine, Weiss Memorial Hospital, Chicago, IL, USA; 2 Department of Medicine, Section of Cardiology, University of Chicago Medical Center, Chicago, IL, USA

## Abstract

A 27 year-old man who presented to the hospital with progressive lower extremity weakness, developed an acute ST elevation myocardial infarction on his second hospital day. Primary angioplasty to the left anterior descending coronary artery was performed. Due to persistent dyspnea, the patient underwent a diagnostic chest computed tomography which confirmed multiple small pulmonary emboli. Laboratory analysis revealed a megaloblastic anemia with a reduced vitamin B12 level and positive titers for antibodies against intrinsic factor, establishing a diagnosis of pernicious anemia. Screening for hypercoaguable markers documented an isolated severely elevated homocysteine levels (105 μmol/l). No other significant risk factors for coronary artery disease including a family history of premature atherosclerosis were identified. This case illustrates the importance of testing for hyperhomocysteinemia as part of a workup for atherothrombotic disease, especially in patients without other significant risk factors. The severity of hyperhomocysteinemia found in our patient is unusual for patients with vitamin B12 malabsorption and raises the question of whether the widely practiced folic acid fortification in the United States may mask or even worsen vitamin B12 deficiency over time, leading to more severe cases of vitamin B12 deficiency and hyperhomocysteinemia than seen in the past.

## Background

Multiple epidemiological studies have demonstrated a strong association between atherosclerotic vascular diseases and hyperhomocysteinemia [1-3]. Levels of hyperhomocysteinemia have been classified into mild-moderate (homocysteine level, 16 to 30 μmol per liter), intermediate (>31 to 100 μmol per liter), and severe (>100 μmol per liter). Meta-analysis calculated an odds ratio of 1.32 (95% confidence interval 1.19 to 1.45) for the presence of ischemic heart disease with a 5 μmol/l increase in serum homocysteine [4,5]. The independent risk of cardiovascular events conferred by moderately elevated serum homocysteine was the rational behind the design for several recent prospective and randomized clinical trials administering folic acid and B-vitamin supplementations to lower serum homocysteine levels; all studies to-date, however, have failed to achieve improved vascular outcomes [6-10].

While mild to moderately elevated homocysteine levels are caused by a variety of factors including nutritional deficiency of vitamin cofactors involved in the homocysteine metabolism, severe elevation is generally only observed in rare cases of genetic defects in the enzymes which metabolize homocysteine. We present a young man with an acute myocardial infarction due to a thrombotic occlusion of the left anterior descending coronary artery who also developed pulmonary emboli in the setting of severe hyperhomocysteinemia secondary to pernicious anemia.

## Case presentation

**Figure 1:**
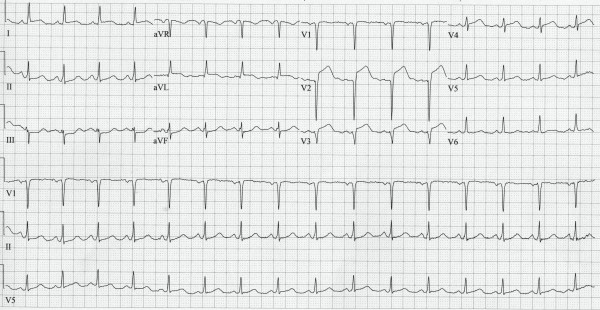
**Electrocardiogram: ST-elevations in lead V1–V4 and I and aVL with reciprocal ST-depression in the inferior leads III and aVF**.

**Figure 2:**
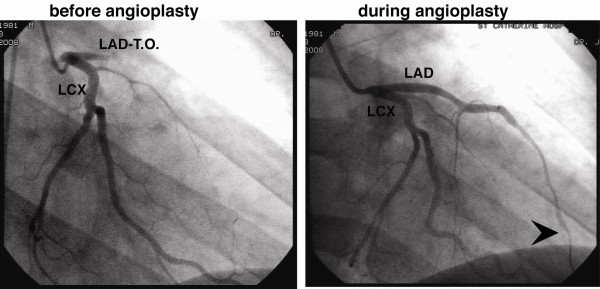
**Coronary angiogram showing a total occluded left anterior descending artery (LAD-T.O.) and a normal left circumflex coronary artery (LCX)**. Angioplasty restored TIMI II flow with distal filling defects due to residual thrombus (arrow).

A 27 year-old male was admitted to the hospital with a one month history of progressive and severe weakness, numbness, and paresthesias of bilateral lower extremities. The initial physical examination revealed normal vital signs, an unremarkable cardiovascular examination and disabling weakness in the lower extremities with impaired vibratory sensation and absent reflexes in both ankles. On his second day of hospitalization, the patient suddenly developed crushing retrosternal chest pain, shortness of breath and excessive diaphoresis. The patient was anxious, tachypnic and his blood pressure at this time was 80/60 mmHg. An electrocardiogram (Figure 1) revealed anteroseptal ST-segments elevations with reciprocal changes involving the inferior leads, consistent with an acute myocardial infarction. A coronary angiogram (Figure 2) was subsequently performed showing a large occluding thrombus in the proximal left anterior descending artery (LAD). The left circumflex and right coronary arteries were patent without prominent luminal irregularities. The LAD was successfully revascularized with percutaneous balloon angioplasty with immediate improvement in the patient's hemodynamics. The patient was started on aspirin 325 mg daily, therapeutic unfractionated Heparin, metoprolol and lisinopril. A transthoracic echocardiogram, completed the day after the intervention, revealed hypokinesis of the apical anterior wall and mildly reduced left ventricular function with an estimated ejection fraction of 45%. The right ventricular size and function were normal. Despite the successful coronary intervention, the patient continued to experience dyspnea. Therefore, a helical computed tomography (CT)-scan of the chest was performed which showed multiple small pulmonary emboli to the left lower lung. Doppler ultrasound of the lower extremities did not show deep venous thrombosis.

An extensive laboratory evaluation for his neuropathy as well as hypercoagualble state revealed a mild anemia with significant macrocytosis (hemoglobin 11.2 g/dl, mean corpuscular volume 122 fl), hypersegmented neutrophils (8.5%) and a high lactate dehydrogenase (LDH) (552 U/L – Normal <180). Serum vitamin B12 blood levels were low at158 pg/ml (Normal 197–894) with a normal serum folate level of 12.1 ng/ml (Normal >3). Homocysteine levels were severely elevated to105 μmol/l. Genetic testing for the methylenetetrahydrofolate reductase C677T gene mutation was negative. All other tests done to identify a hypercoagulable state were normal as summarized in Table 1. The patient tested positive for anti-intrinsic factor antibodies (IFBA quantitative result of 6.61 arbitrary units, Mayo Clinic, Rochester MN) which confirmed a diagnosis of pernicious anemia; the patient was started on daily intramuscular vitamin B12 injections. His lipid profile was normal (total cholesterol of 139 mg/dl, LDL 51 mg/dl, HDL 40 mg/dl and triglycerides of 75 mg/dl).

**Table 1:** Summary of thrombophilia screening laboratory findings

**Tests**	**Results (Normal Range)**
Prothrombin Time	10.6 seconds (8.9–11.7)

International Normalized Ratio (INR)	1 (0.8–1.3)

Activated Partial Thromboplastin Time	25.1 (24.5–35.2)

D-Dimer	6.79 mg/l (0.97–2.12)

Protein C activity (%)	110% (111–173)

Protein S activity (%)	118% (66–173)

Antithrombin III activity	110% (88–130)

Factor V Leiden mutation	Negative

Prothrombin gene mutation	Negative

Phospholipid (Cardiolipin) AB IgG	Negative with <0.4 (norm <10.0)

Phospholipid (Cardiolipin) AB IgM	Negative with <0.4 (norm <10.0)

Phospholipid (Cardiolipin) AB IgA	Negative with <0.4 (norm <10.0)

Lupus Anticoagulant	Absent

DNA doublestranded AB IgG	Negative with <0.4 (norm <10.0)

A repeat transthoracic echocardiogram performed seven days after the myocardial infarction showed persistent anterior hypokinesis with normal left ventricular function. The right ventricular size and function were again documented to be normal. After ten days of treatment with vitamin B12 and folic acid supplements, homocysteine levels normalized to 12.9 μmol/l. His neurological symptoms gradually improved and the patient was ultimately transferred to a physical therapy rehabilitation facility with the following medical therapy: warfarin, vitamin B12 s.c. supplementation, aspirin, metoprolol, lisinopril and eplerenone.

## Discussion

Kilmer McCully, the first pioneer to study the role of hyperhomocysteinemia in the pathogenesis of arteriosclerosis, began by studying two children with two different forms of hereditary enzymatic defects which led to severe hyperhomocystenemia. Both these children presented with significant vascular disease at very young ages. In 1969, McCully concluded that the vascular damage in both cases was caused by an severely elevated homocysteine level and he suggested a possible similar mechanism in patients without genetic defects but who have mild to moderately elevated homocysteine levels [11]. In the years that followed, a large number of studies supported the presence of a strong association between elevated homocysteine levels and various atherothrombotic diseases including stroke, venous thromboembolism (VTE) and ischemic cardiac disease [1-3]; a meta-analysis of clinical observational studies consistently found that a 25 percent reduction in the serum homocysteine concentration is associated with an 11 percent lower risk of ischemic heart disease (odds ratio, 0.89; 95% confidence interval, 0.83 to 0.96) and a 19 percent lower risk of stroke (odds ratio, 0.81; 95% confidence interval, 0.69 to 0.95) [5]. Furthermore, *in vitro *studies and animal models of hyperhomocysteinemia have clearly shown multiple mechanisms of homocysteine induced vascular dysfunction. Inhibition of endothelial cell nitric oxide production, enhanced oxidative stress, IL-8 up-regulation leading to enhanced leukocyte recruitment, and up-regulation of tissue factor within the vasculature are among many mechanisms proposed underpinning homocysteine induced cell injury [12-15]. However, lowering mildly elevated homocysteine levels in patients with and without vascular disease using vitamin supplementation did not show a reduction in cardiovascular events in several prospective and randomized clinical studies [7-10]. Possibly harmful effects of vitamin therapy in certain settings have been suggested in some trials. For example, the folate after coronary intervention (FACIT) trial demonstrated that administration of folic acid, vitamin B12 and vitamin B6 after coronary stenting might increase the risk of in-stent restenosis and the need for target-vessel revascularization [16].

In contrast to those studies which included patients with mild to moderate elevations in homocysteine, our patient had a severely elevated homocysteine level. Interestingly, the etiology of the severity of hyperhomocysteinemia in this case is not exactly clear as isolated vitamin B12 deficiency due to pernicious anemia has not been reported to directly cause such unusually high homocysteine levels [17-19]. We did not identify other causes of hyperhomocysteinemia and genetic testing for the common C677T MTHFR gene mutation was negative. We realize the limitation of testing for only one gene mutation, given that a number of other genetic mutations in enzymes involved in homocysteine metabolism can contribute to an elevation in plasma homocysteine levels [20]. However, the absence of a family history for atherothrombotic diseases together with the normalization of his homocysteine level after only ten days of parenteral vitamin B12 supplementation strongly support that indeed vitamin B12 malabsorption secondary to pernicious anemia is the underlying cause of his severe hyperhomocysteinemia. His normal folate level likely may have contributed to the severity of the homocysteine elevation by masking his megaloblastic anemia, and leading to a later presentation of a more severe neuropathy.

In fact, a recent study using data from the National Health and Nutrition Survey (NHANES) between 1999–2002 found that participants with vitamin B12 deficiency and high serum folate had increased homocysteine levels compared to participants who had the combination of vitamin B12 deficiency and low serum folate, suggesting a role of folate levels in vitamin B12's enzymatic functions [21]. We speculate that his normal folate levels may have contributed to the delay in diagnosing pernicious anemia leading to severe hyperhomocysteinemia and the consequent development of vascular injury and hypercoaguability which presented as an acute myocardial infarction and multiple pulmonary emboli.

## Conclusion

This case clearly demonstrates that vitamin B12 deficiency caused by pernicious anemia may lead to a severely elevated homocysteine levels that can be rapidly corrected with vitamin B12 supplements. Severe hyperhomocysteinemia can be a risk factor for endothelial damage, vascular inflammation and thrombus formation leading to pulmonary emboli and in-situ coronary artery thrombus formation. We conclude that severe hyperhomocysteinemia needs to be considered in the work-up of acute myocardial infarction not explained by atherosclerotic heart disease. Although studies have shown no preventive benefit from correcting mildly elevated homocysteine levels, severe elevation in homocysteine level should be corrected with appropriate vitamin therapy to prevent vascular complications.

## Abbreviations

CT: Computed Tomography; MI: Myocardial Infarction; LAD: Left Anterior Descending artery.

## Consent

Written informed consent was obtained from the patient for publication of this case report and any accompanying images. A copy of the written consent is available for review by the Editor-in-Chief of this journal.

## Competing interests

The authors declare that they have no competing interests.

## Authors' contributions

AM collected the data and drafted the manuscript. AD and MAH coordinated and edited the manuscript. All authors read and approved the final manuscript.
